# Multidimensional Radiological Assessment of Delirium in the Emergency Department

**DOI:** 10.3390/healthcare13151871

**Published:** 2025-07-31

**Authors:** Alberto Francesco Cereda, Claudia Frangi, Matteo Rocchetti, Andrea Spangaro, Lorenzo Tua, Antonio Gabriele Franchina, Matteo Carlà, Lucia Colavolpe, Matteo Carelli, Anna Palmisano, Massimiliano Etteri, Stefano Lucreziotti

**Affiliations:** 1 Cardiology Unit, ASST Santi Paolo e Carlo, 20153 Milan, Italy; matteo.rocchetti@unimi.it (M.R.); andrea.spangaro1@gmail.com (A.S.); lorenzo.tua1@gmail.com (L.T.); gfranchina.ct@gmail.com (A.G.F.); matteo.carla@asst-santipaolocarlo.it (M.C.); stefano.lucreziotti@asst-santipaolocarlo.it (S.L.); 2 Emergency Department, ASST Santi Paolo e Carlo, 20153 Milan, Italy; claudia.frangi93@gmail.com (C.F.); luciacolavolpe@libero.it (L.C.); teo26@hotmail.it (M.C.); massimiliano.etteri@asst-santipaolocarlo.it (M.E.); 3 Experimental Imaging Center, IRCCS San Raffaele Scientific Institute, 20132 Milan, Italy; palmisano.anna@hsr.it; 4 School of Medicine, Vita-Salute San Raffaele University, 20132 Milan, Italy

**Keywords:** delirium, coronary calcium score, cachexia, brain atrophy, mortality

## Abstract

**Background:** Delirium is a common, underdiagnosed neuropsychiatric syndrome in older adults, associated with high mortality and functional decline. Given its multifactorial nature and overlap with frailty, radiological markers may improve risk stratification in the emergency department (ED). **Methods:** We conducted a retrospective study on a small sample of 30 patients diagnosed with delirium in the emergency department who had recently undergone brain, thoracic, or abdominal CT scans for unrelated clinical indications. Using post-processing software, we analyzed radiological markers, including coronary artery calcifications (to estimate vascular age), cerebral atrophy (via the Global Cortical Atrophy scale), and cachexia (based on abdominal fat and psoas muscle volumetry). **Results**: Five domains were identified as significant predictors of 12-month mortality in univariate Cox regression: vascular age, delirium etiology, cerebral atrophy, delirium subtype (hyperactive vs. hypoactive), and cachexia. Based on these domains, we developed an exploratory 10-point delirium score. This score demonstrated acceptable diagnostic accuracy for mortality prediction (sensitivity 0.93, specificity 0.73, positive predictive value 0.77, negative predictive value 0.91) in this limited cohort. **Conclusions:** While preliminary and based on a small, retrospective sample of 30 patients, this multidimensional approach integrating clinical and radiological data may help improve risk stratification in elderly patients with delirium. Radiological phenotyping, particularly in terms of vascular aging and sarcopenia/cachexia, offers objective insights into patient frailty and could inform more personalized treatment pathways from the ED to safe discharge home, pending further validation.

## 1. Introduction

Delirium is a frequently underdiagnosed acute neuropsychiatric syndrome characterized by sudden changes in mental status with an interplay of medical, environmental, and psychological factors [1,2].

Delirium is characterized by fluctuating disturbances in consciousness and cognitive function. Unlike dementia, it has a rapid onset and is potentially reversible. In the emergency department, delirium is a condition associated with increased morbidity, mortality, and length of hospital stay.

Among older adults, there is a substantial overlap between delirium, dementia, and clinical frailty, conditions that share predisposing factors, and adverse outcomes. Delirium may thus present as an acute exacerbation in patients with underlying dementia or serve as a sentinel marker of clinical vulnerability.

It typically presents with fluctuating levels of consciousness, transient memory impairment, psychomotor disturbances, and disruption of the sleep–wake cycle [3]. Despite its high prevalence, delirium often goes unrecognized, particularly in emergency settings, where up to 95% of cases may be missed, leading to significant negative consequences for patient outcomes [4].

The condition is strongly associated with aging, frailty, multimorbidity, and baseline cognitive impairment and is a well-established predictor of long-term cognitive and functional decline—even after hospital discharge [5].

Currently, the diagnosis relies primarily on clinical tools such as the Confusion Assessment Method (CAM), which identifies delirium through features including altered consciousness, symptom fluctuation, disorganized thinking, and perceptual disturbances [6].

Management of delirium requires prompt recognition of the underlying cause. Initial treatment strategies prioritize non-pharmacological interventions, such as environmental reorientation, cognitive stimulation, and sleep hygiene, while pharmacological approaches are reserved for severe or refractory cases [7].

However, delirium is not merely a transient issue, it is associated with serious long-term complications, including falls, aspiration pneumonia, institutionalization, disability, and both short- and long-term mortality [8,9].

Recent evidence highlights frailty as a major determinant of vulnerability to delirium and its complications. In this context, radiological parameters have increasingly been used to estimate biological age and assess frailty, serving as valuable prognostic markers in various clinical scenarios [10].

Given the overlapping pathophysiology between frailty, neurocognitive dysfunction, and delirium, we hypothesized that CT-derived radiological markers could provide additional prognostic insights. In particular, we investigated whether imaging such as vascular calcification (as a surrogate for vascular aging), brain atrophy, and muscle wasting (cachexia) were associated with the risk and outcome of delirium.

By integrating clinical and imaging data, we aimed to propose a simple, multiparametric scoring system to improve prognostic stratification and help guide care in elderly patients with delirium presenting to the emergency department, facilitating care pathways for an acute condition with long-term chronic implications.

## 2. Methods

### 2.1. Purpose of the Study

The primary objective of this study was to analyze a cohort of patients admitted to the emergency department with a diagnosis of delirium and to identify predictors of mortality through the development of a multiparametric score.

### 2.2. Inclusion Criteria

We included 30 patients admitted to the Emergency Department of San Carlo Borromeo Hospital in Milan, Italy. The diagnosis of delirium was made according to the Confusion Assessment Method (CAM) [11].

Patients were classified based on the underlying etiology of delirium into the following categories: neurological, septic, cardiac–hypoxic, and metabolic. Each delirium case was also categorized as either hyperactive or hypoactive.

To be included in the study, patients were required to have undergone a recent brain, chest, and abdominal CT scan. All three imaging exams had to have been performed for clinical purposes within 90 days prior to the diagnosis of delirium. Clinical history and demographic data were collected, and patients were followed up to assess clinical outcomes.

### 2.3. Study Design

This was a retrospective cohort study conducted at San Carlo Borromeo Hospital in Milan (2023–2024). The study was conceived as a pilot study using innovative clinical–radiological markers in a frail, understudied population

### 2.4. Outcomes

The primary outcome was all-cause mortality at 12 months. Mortality data were obtained from the administrative health database of the Lombardy “Regional Health Record—Fascicolo Sanitario Regionale”.

### 2.5. Radiological Variables

Radiological analysis was performed using Synapse 3D (Fujifilm) v3.0 post-processing software [12]. This allowed for the assessment of the following:

- Cardiovascular calcifications;
- Body composition (visceral and abdominal fat);
- Psoas muscle volume (as an estimate of sarcopenia/cachexia).

To estimate vascular age, we used the MESA Risk Score and Coronary Age Calculator, assigning a higher vascular age to patients whose calcium-derived age exceeded their chronological age [13,14].

Cachexia was defined using the median values of visceral fat and psoas muscle volume within the study population. The cut-off for psoas muscle volume (in mm^3^) used to define cachexia was based on the median value within our study population, given the absence of standardized thresholds in this setting. We acknowledge this as a study-specific definition that limits external validation.

Brain CT scans were reviewed by an experienced neuroradiologist and classified as mild, moderate, or severe according to the Global Cortical Atrophy (GCA) scale, also known as the Pasquier scale. The variable was analyzed as a categorical variable.

### 2.6. Statistical Analysis

Descriptive statistics summarized baseline demographic, clinical, and radiological characteristics. Continuous variables were reported using mean ± standard deviation or median (interquartile range, IQR), and categorical variables using counts and percentages. In particular, radiological quantitative variables, such as calcium score or anthropometric measurements, were reported as median and IQR due to their non-normal distribution.

Comparisons between groups (e.g., survivors vs. non-survivors; hyperactive vs. hypoactive delirium) were performed using the Student’s *t*-test or Mann–Whitney U test for continuous variables, and the chi-square or Fisher’s exact test for categorical variables, as appropriate.

Univariate Cox proportional hazards regression analysis was performed to identify predictors of 12-month mortality. Given the small sample size and the limited number of events (15 deaths), we chose to forgo multivariate analyses to avoid model overfitting and unstable estimates. The generally accepted rule of thumb recommends at least 10 events per predictor variable in survival analysis, which was not feasible in our cohort.

**Figure 1:**
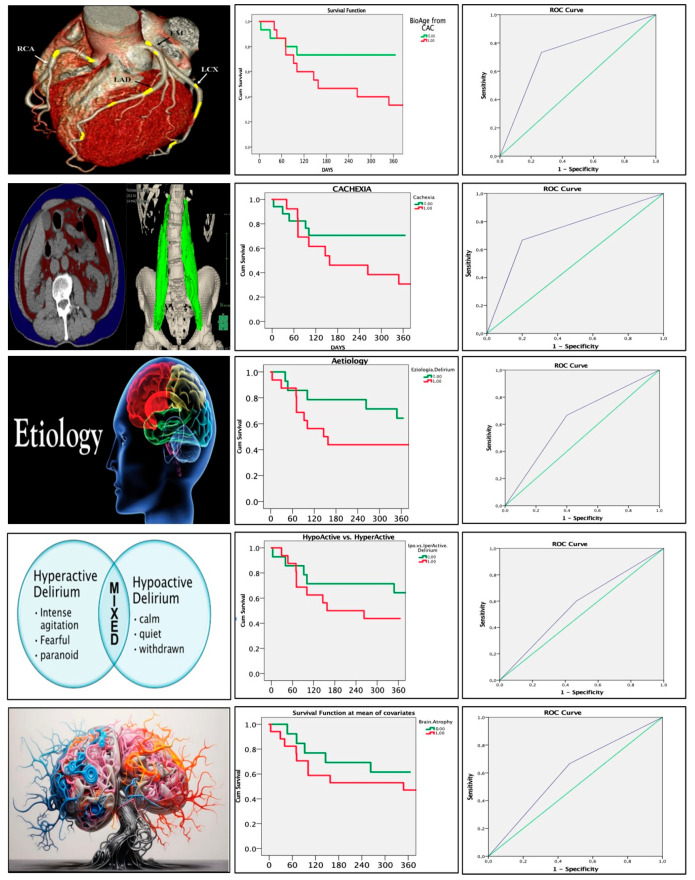
ROC and univariate Cox regression of the 5 variables included in the delirium score.

Significant variables were incorporated into a multiparametric delirium score. The diagnostic performance of the score was evaluated using ROC curve analysis (see the third column of Figure 1), with calculation of sensitivity, specificity, positive predictive value (PPV), and negative predictive value (NPV). A two-sided *p*-value < 0.05 was considered statistically significant. All analyses were conducted using IBM SPSS Statistics, Version 23.0 (IBM Corp., Armonk, NY, USA).

## 3. Results

### 3.1. Population Characteristics

A total of 30 patients were included in the study. The mean age was 85 ± 5 years, and 43.3% were female (n = 13). The etiology of delirium was classified as septic (30%, n = 9), neurological (33.3%, n = 10), cardiac–hypoxic (20%, n = 6), or metabolic (16.7%, n = 5). Hypoactive delirium was more common (53.3%, n = 16) than hyperactive delirium (46.7%, n = 14). (See Table 1, Table 2 and Table 3)

### 3.2. Etiological Subgroups

- **Septic Delirium (n = 9):** Mean age 84 ± 3 years; 11.1% female. Hypoactive in 44.4%, hyperactive in 55.6%. Mean SBP 128 ± 17 mmHg, DBP 75 ± 10 mmHg, HR 93 ± 10 bpm. Charlson Index: 8 ± 3. One-year mortality: 33.3% (n = 3).
- **Neurological Delirium (n = 10):** Mean age 86 ± 5 years; 50% female. Hypoactive 60%, hyperactive 40%. SBP 127 ± 25 mmHg, DBP 71 ± 13 mmHg, HR 83 ± 18 bpm. Charlson Index: 7 ± 3. One-year mortality: 70% (n = 7).
- **Cardiac–hypoxic Delirium (n = 6):** Mean age 83 ± 7 years; 50% female. Hypoactive and hyperactive equally distributed (50%). SBP 151 ± 39 mmHg, DBP 86 ± 18 mmHg, HR 89 ± 7 bpm. Charlson Index: 6 ± 1. One-year mortality: 50% (n = 3).
- **Metabolic Delirium (n = 5):** Mean age 87 ± 4 years; 80% female. Hypoactive 60%, hyperactive 40%. SBP 153 ± 21 mmHg, DBP 79 ± 12 mmHg, HR 83 ± 4 bpm. Charlson Index: 7 ± 2. One-year mortality: 40% (n = 2).

### 3.3. Clinical History and Outcomes

Cognitive impairment was reported in 40% of patients (n = 12), functional disability in 53.3% (n = 16), anemia in 33.3% (n = 10), and electrolyte abnormalities in 26.7% (n = 8). The average hospital stay was 18 ± 15 days. Mortality rates were as follows:

- 30-day: 7% (n = 2);
- 3-month: 13% (n = 4);
- 6-month: 40% (n = 12);
- 1-year: 50% (n = 15).

### 3.4. Hyperactive vs. Hypoactive Delirium

No significant differences were observed in mean age (86 ± 3 vs. 84 ± 6 years, *p* = ns) or gender distribution between hyperactive (n = 14) and hypoactive (n = 16) delirium groups. The Charlson Comorbidity Index was higher in the hypoactive group (8 ± 2 vs. 6 ± 2, *p* = 0.07).

Estimated 10-year survival was significantly higher in the hyperactive group (25%) compared with the hypoactive group (6%, *p* = 0.013). One-year mortality: 43% in the hyperactive group vs. 50% in the hypoactive group (*p* = ns) (See Table 3).

### 3.5. Radiological Parameters and Mortality

No significant differences were found in coronary artery calcium scores (mm^3^) between survivors and non-survivors (50 ± 122 vs. 54 ± 87, *p* = ns). Similarly, abdominal fat and visceral fat were comparable.

Psoas muscle volume showed a trend toward significance (survivors: 211 ± 70 vs. non-survivors: 168 ± 41, *p* = 0.054). Cachexia was significantly more prevalent in non-survivors (66.6%) compared with survivors (20%, *p* = 0.025). (See Table 4).

### 3.6. Delirium Score and ROC Analysis

ROC curve analysis identified cachexia and vascular aging as the strongest predictors of 12-month mortality (AUC = 0.733 for both).

Based on predictive weight, the delirium score was constructed as follows:

- Cachexia: 3 points;
- Vascular aging: 3 points;
- Etiology of delirium: 2 points;
- Delirium type: 1 point;
- Brain atrophy: 1 point.

**Figure 2:**
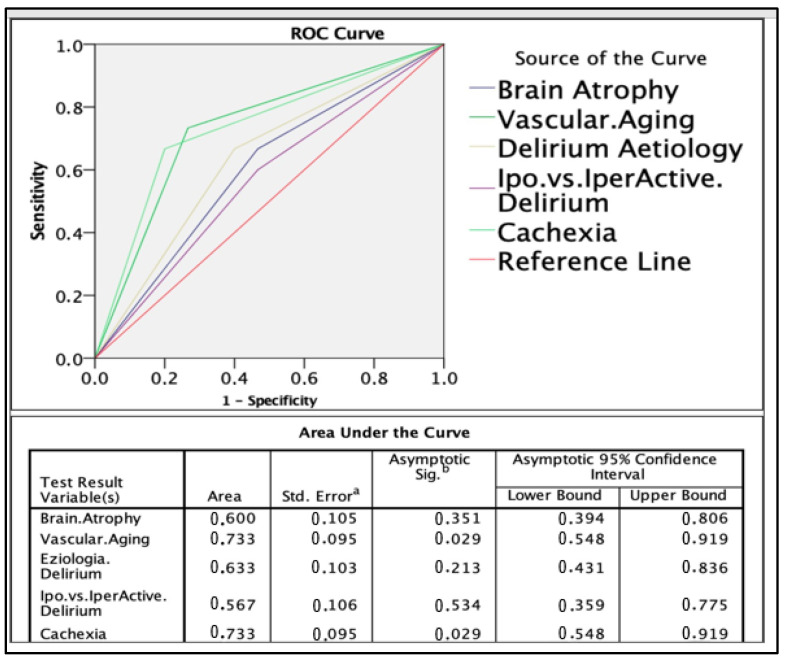
Comparison of ROC curves of the 5 variables. Notes: “a” refers to the method used to calculate the standard error (typically under a nonparametric assumption). “b” refers to the null hypothesis tested for the AUC (i.e., true area = 0.5).

**Figure 3:**
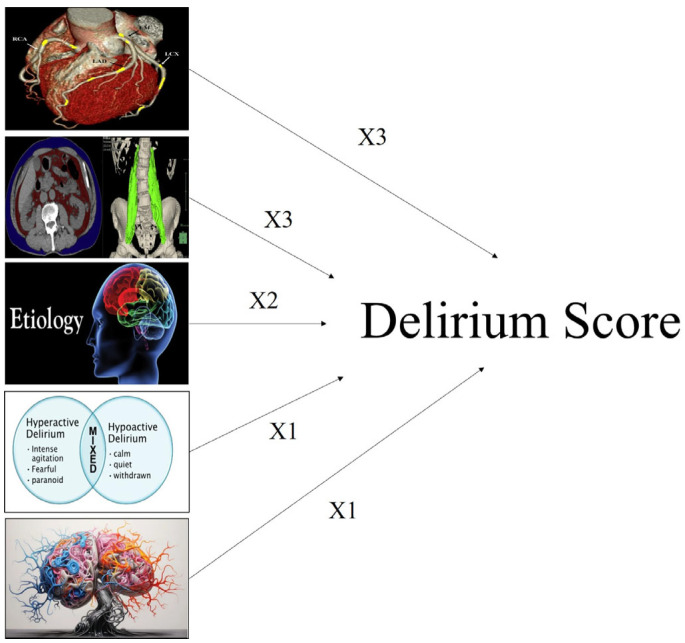
Importance and weight of the individual variables in the score calculation.

**Figure 4:**
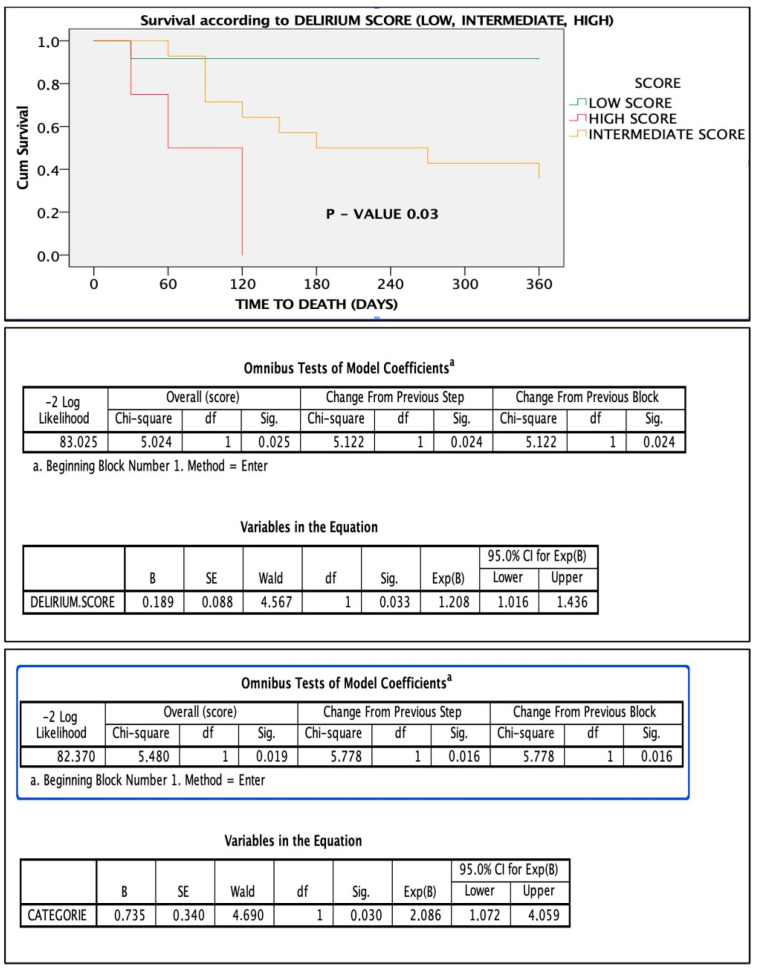
Survival based on point score: low score vs. intermediate scores vs. high score. Below is the figure statistical significance of the score in the Cox regression model.

**Figure 5:**
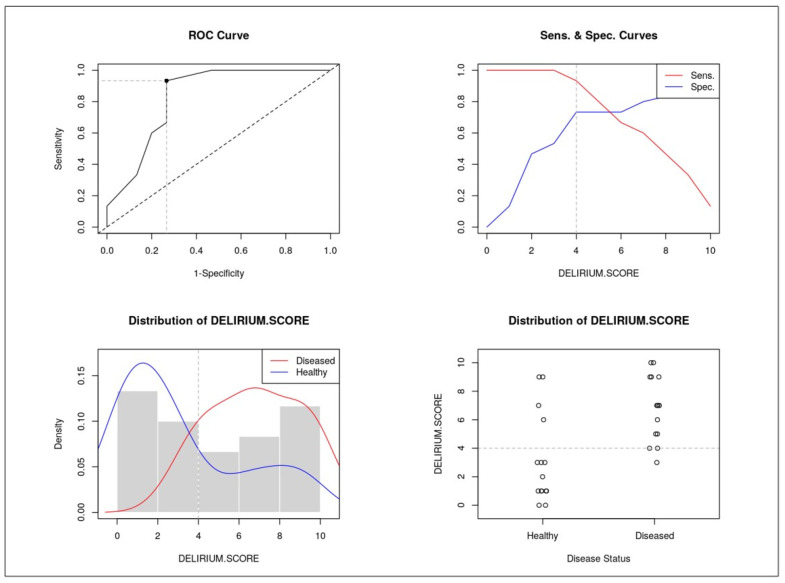
ROC curve, sensitivity, and specificity of the score.

**Figure 6:**
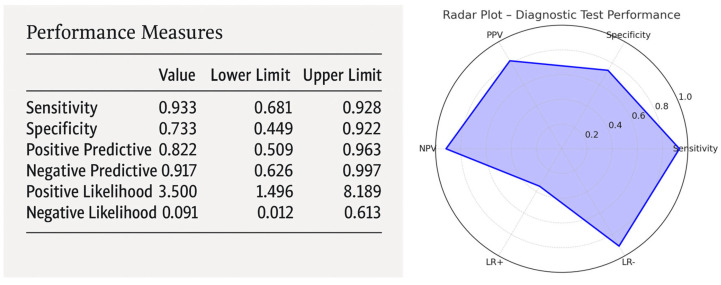
Statistical performance of the score in predicting mortality.

The score stratified patients into low-, intermediate-, and high-risk groups. Patients with a score > 4 had significantly higher 1-year mortality (Figure 1, Figure 2, Figure 3, Figure 4, Figure 5 and Figure 6).

## 4. Discussion

This study provides novel insights into the clinical and radiological features of delirium in elderly patients, emphasizing distinct outcomes based on delirium subtypes, etiologies, and mortality predictors. Our findings confirm that delirium, particularly in frail older adults, is associated with poor prognoses, including a high 1-year mortality rate, especially in cases of neurological delirium [15,16]. The study also highlights the value of a multiparametric delirium score to improve mortality prediction, potentially supporting more informed clinical decision-making.

### 4.1. Delirium Etiologies and Mortality

Our results demonstrate a clear association between the etiology of delirium and patient outcomes. Neurological delirium was linked to the highest 1-year mortality (70%), followed by cardiac–hypoxic delirium (50%). In contrast, septic delirium had a comparatively lower mortality rate (33%). These findings are consistent with existing literature [15,16], showing increased mortality in delirium of neurological origin. This may be due to underlying brain dysfunction, which predisposes patients to cognitive deterioration and reduced cerebral reserve.

### 4.2. Hyperactive vs. Hypoactive Delirium

Differentiating between hyperactive and hypoactive delirium is clinically relevant, as these subtypes carry distinct prognostic implications [17]. In our cohort, patients with hyperactive delirium had significantly higher estimated 1-year survival than those with hypoactive delirium (25% vs. 6%, *p* = 0.013). This supports prior evidence that hypoactive delirium is frequently underrecognized and is associated with worse outcomes, possibly due to delayed diagnosis and intervention. The higher Charlson Comorbidity Index observed in the hypoactive group (8 ± 2 vs. 6 ± 2, *p* = 0.07) may reflect a greater baseline frailty and multimorbidity, further contributing to their poorer prognosis.

### 4.3. Radiological Features as Prognostic Indicators

Although coronary calcification, visceral fat, and psoas volume did not differ significantly between survivors and non-survivors, the trend toward significance in psoas volume (*p* = 0.054) is noteworthy. Sarcopenia, as suggested by reduced psoas muscle volume, is increasingly recognized as a marker of frailty and adverse outcomes in geriatric populations [18]. Moreover, the significantly higher prevalence of cachexia among non-survivors (66.6% vs. 20%, *p* = 0.025) highlights the prognostic impact of malnutrition and muscle wasting. To our knowledge, there is no universally accepted cut-off in the literature for psoas muscle volume (mm^3^) to define cachexia, particularly using 3D volumetric measurements. Given this lack of standardized thresholds, we used the median value of our study cohort to dichotomize cachexia status, acknowledging this as a study-specific definition that limits external validity. Compared with survivors, deceased patients exhibited a trend toward higher coronary and valvular calcifications across all measured sites, although none of these differences reached statistical significance. Conversely, significant disparities were observed in body composition: deceased patients had a markedly lower psoas muscle volume, suggesting sarcopenia, and tended to have reduced abdominal fat percentages. Visceral fat was also lower in the deceased group, though not significantly. These findings point to a potential association between adverse outcomes and a catabolic body profile characterized by diminished muscle mass and reduced fat reserves.

### 4.4. The Delirium Score: Improving Mortality Prediction

One of the key contributions of this study is the development of a delirium score integrating five variables: brain atrophy, vascular aging, delirium etiology, delirium subtype, and cachexia. Cachexia and vascular aging were the strongest predictors, each contributing three points (AUC = 0.733). By combining radiological and clinical parameters, this composite score demonstrated improved predictive performance compared with individual metrics. This supports a holistic approach to understanding delirium as a multifactorial syndrome, where prognosis depends on the interaction of systemic, cognitive, and functional vulnerability.

### 4.5. Clinical Implications

Our findings have practical implications. Early identification and targeted management of high-risk delirium cases, especially the hypoactive subtype, are essential to improving outcomes [19,20]. Given the high mortality associated with neurological and frailty-related delirium, a multidisciplinary approach is warranted. This includes early neurological evaluation, nutritional interventions, and physical rehabilitation. The proposed delirium score may serve as a simple, clinically useful tool for risk stratification, supporting prognosis estimation and shared decision-making in emergency and geriatric care.

### 4.6. Study Limitations

This study has several limitations. First, the sample size is small, limiting the generalizability of the findings. Larger, multicenter studies are necessary to validate the delirium score and assess its reproducibility. Our focus was limited to clinical and radiological parameters; biochemical markers (e.g., inflammatory or neurodegenerative indicators) were not included. Future research should aim to integrate these additional variables into the score to enhance its predictive power. Small sample size (n = 30) limits the robustness and generalizability of the proposed prognostic score. The absence of a non-delirium control group reduces the ability to distinguish delirium-specific predictors from general frailty markers, and inclusion criteria may introduce selection bias. Additionally, the lack of external validation and omission of multivariable Cox regression limit control for confounding factors.

### 4.7. Future Perspectives

Radiological imaging contains a substantial amount of prognostic information that remains largely unexplored. Indeed, it represents a rich yet underexploited source of prognostic data that could enhance risk stratification and guide personalized management strategies. Dedicated post-processing of routinely acquired CT scans could offer new, scalable tools for frailty and delirium assessment. For example, real-time classification of brain atrophy using artificial intelligence may help phenotype delirium more accurately in the emergency department. Similarly, early identification of sarcopenia or visceral fat changes could guide tailored treatment pathways.

As Thomas Sydenham famously said, “*Man is as old as his arteries*”. Coronary artery calcium, when assessed even from non-gated scans, can serve as a surrogate for vascular age. In our study, radiological analysis does not aim to replace clinical evaluation but rather to provide standardized, objective parameters to complement the phenotyping of delirium.

Importantly, the score we propose is versatile. Radiological variables could be replaced or complemented by clinical equivalents—for instance, cardiovascular comorbidities in place of vascular age, or a documented history of dementia in place of imaging-defined brain atrophy. Further validation in larger and external cohorts will be essential to confirm its utility.

It is likely that the data we have painstakingly collected through patient-by-patient, exam-by-exam post-processing will soon be acquired with just a few clicks using artificial intelligence software. Radiological phenotyping, when applied to the clinical context of delirium, may facilitate the identification of specific patient subgroups who could benefit from targeted treatments, an especially promising prospect for a condition that currently lacks effective therapeutic options.

## 5. Conclusions

In conclusion, this study highlights the complexity of delirium in elderly patients and its strong association with mortality. Delirium outcomes vary based on subtype, etiology, and underlying frailty. We propose a novel, multiparametric delirium score that integrates clinical and radiological data to improve prognostic accuracy. Cachexia and vascular aging emerged as the most influential predictors of 1-year mortality. Early identification, comprehensive management, and stratified care approaches, particularly for hypoactive delirium, may improve survival in this vulnerable population. Further research is warranted to validate these findings and refine the score.

## Figures and Tables

**Table 1:** Clinical profile of the delirium population.

Number of patients, N	30
Average age, years ± SD	85 ± 5
Female sex, n/N (%)	13 (43.3)
**Delirium etiology**	
Cardiac/hypoxic, n/N (%)	6 (20%)
Septic, n/N (%)	9 (30%)
Neurological, n/N (%)	10 (33.3%)
Metabolic, n/N (%)	5 (16.7%)
**Delirium type**	
Hypoactive, n/N (%)	16 (53.3%)
Hyperactive, n/N (%)	14 (46.7%)
Systolic BP, mmHg ± SD	136 ± 27
Diastolic BP, mmHg ± SD	77 ± 14
Heart rate, bpm ± SD	88 ± 13
Charlson Comorbidity Index, N ± SD	7 ± 2
Cognitive decline history, n/N (%)	12 (40%)
Disability, n/N (%)	16 (53.3%)
Anemia history, n/N (%)	10 (33.3%)
Electrolyte abnormalities, n/N (%)	8 (26.7%)
Length of stay, days ± SD	18 ± 15
**Mortality**	
30-day mortality, n/N (%)	2 (7%)
3-month mortality, n/N (%)	7 (13%)
6-month mortality, n/N (%)	12 (40%)
1-year mortality, n/N (%)	15 (50%)

**Table 2:** Clinical profile based on the etiology of delirium.

	Septic (1)	Neurological (2)	Cardiac–Hypoxic (3)	Metabolic (4)
Number of patients, N	9	10	6	5
Average age, years ± SD	84 ± 3	86 ± 5	83 ± 7	87 ± 4
Female sex, n/N	1	5	3	4
Hypoactive, n/N	4	6	3	3
Hyperactive, n/N	5	4	3	2
Systolic BP, mmHg ± SD	128 ± 17	127 ± 25	151 ± 39	153 ± 21
Diastolic BP, mmHg ± SD	75 ± 10	71 ± 13	86 ± 18	79 ± 12
Heart rate, bpm ± SD	93 ± 10	83 ± 18	89 ± 7	83 ± 4
Charlson Comorbidity Index, N ± SD	8 ± 3	7 ± 3	6 ± 1	7 ± 2
Cognitive decline history, n/N	3	4	4	1
Disability, n/N	6	5	3	2
Anemia, n/N	3	6	0	1
Electrolyte abnormalities, n/N	0	2	2	4
Length of stay, days ± SD	23 ± 13	20 ± 18	16 ± 14	7 ± 3
1-year mortality, n/N (%)	3 (33.3%)	7 (70%)	3 (50%)	2 (40%)

**Table 3:** Hyperactive delirium vs. hypoactive delirium.

	Hyperactive	Hypoactive	*p*-Value
Average age, years ± SD	86 ± 3	84 ± 6	ns
Female sex, n/N	6	7	ns
Septic, n/N	5	4	ns
Neurological, n/N	4	6	ns
Cardiac hypoxic, n/N	3	3	ns
Metabolic, n/N	2	3	ns
Systolic BP, mmHg ± SD	139 ± 31	134 ± 24	ns
Diastolic BP, mmHg ± SD	79 ± 15	75 ± 13	ns
Charlson Comorbidity Index, N ± SD	6 ± 2	8 ± 2	0.07
Estimated 10-year survival, %	25	6	0.013
Cognitive decline history, n/N (%)	4 (33.3%)	8 (66.7%)	ns
Disability, n/N	7	9	ns
Anemia, n/N	6	4	ns
Electrolyte abnormalities, n/N	3	5	ns
Length of stay, days ± SD	17 ± 13	18 ± 16	ns
1-year mortality, n/N (%)	6 (43%)	8 (50%)	ns

**Table 4:** Comparison between deceased and survivors.

Radiological Variables	Deceased	Living	All	*p*-Value
Number of patients, N	15	15	30	-
Average age, years ± SD	85 ± 5	84 ± 4	85 ± 5	ns
Common trunk calcifications, median (IQR)	0 (0.02))	0 (0)	0(0)	ns
Anterior descending calcifications, median (IQR)	100 (259)	79 (313)	87 (290)	ns
Circumflex calcifications, median (IQR)	2 (72)	0 (18)	0.75 (27)	ns
Right coronary calcifications, median (IQR)	76 (418)	0 (12)	3.7 (215)	ns
Total calcium score, median (IQR)	300 (711)	100 (724)	192 (838)	ns
Aortic valve calcifications, median (IQR)	0 (71)	0 (0)	0 (35)	ns
Abdominal fat percentage, % median (IQR)	30 (7)	37 (15)	33 (10)	0.06
Visceral abdominal fat, % median (IQR)	30 (10)	35 (18.5)	32 (19)	ns
Psoas volume, mm^3^ ± SD, median (IQR)	150 (40)	200 (41.5)	199 (62)	0.02
Vascular age, years ± SD	83 ± 23	72 ± 16	78 ± 20	ns
Cachexia, n/N (%)	10 (66.6%)	3 (20%)	13 (76%)	0.025
Vascular elder, n/N (%)	11 (73%)	4 (26.6%)	15 (50%)	0.027
Brain atrophy, n/N (%)	10 (66.6%)	7 (46%)	17 (56.6%)	ns
Length of stay, days ± SD	19 ± 17	11 ± 15	18 ± 15	ns

## Data Availability

On request.
